# Supplementary material for: Learning to detect AI texts and learning the limits
Source: PLoS One. 2025 Oct 15;20(10):e0333007. doi: 10.1371/journal.pone.0333007 (PMC12527182; doi:10.1371/journal.pone.0333007)
Supplement: S2 File — All data and scripts used in the study, some additional descriptive statistics, scalable formate figures. (ZIP) [file pone.0333007.s002.tgz › data and scripts/OSF/scripts/analysis/mixed models jamovi/outputs/complete_correctness_nonlearners.pdf]

Generalized Mixed Model

Model Info

Info	Value	Comment
Model Type	Logistic	Model for binary y
Call	glm	Is correct ~ 1 + More readable is human + Confidence + Demographics: age + Demographics: education_ordinal + Demographics: ai_usage_ordinal + Demographics: gender merged + Demographics: field_merged + Reaction time log + GLS Euclidean distance + GLS1 difference + GLS2 difference + GLS3 difference + GLS4 difference + id + Demographics: reading_disorder_merged + GLS8 difference + GLS7 difference + GLS6 difference + GLS5 difference + Demographics: ai_improve + Demographics: ai_risk + Demographics: ai_quality + Demographics: ai_lacks_creativity + Demographics: ai_lacks_understanding + Demographics: ai_grammar + Demographics: ai_avoidance + Demographics: ai_dumbing + Demographics: ai_fascination + (0 + More readable is human + Confidence + Reaction time log + GLS Euclidean distance + GLS1 difference + GLS2 difference + GLS3 difference + GLS4 difference + id + GLS8 difference + GLS7 difference + GLS6 difference + GLS5 difference | Participant ID)
Link function	Logit	Log of the odd of y=1 over y=0
Direction	P(y=1)/P(y=0)	P(Is correct = 1) / P(Is correct = 0)
Distribution	Binomial	Dichotomous event distribution of y
LogLikel.	-1287.871	Unconditional Log-Likelihood
-2*LogLikel.	2575.743	Unconditional absolute deviance
Deviance	1994.258	Conditional relative deviance
R-squared	NaN	Marginal
R-squared	NaN	Conditional
AIC	2851.740	Less is better
BIC	3640.553	Less is better
Residual DF	2106.000	
Chi-squared/DF	0.738	Overdispersion indicator
Converged	yes	
Optimizer	bobyqa	

Note. R-squared cannot be computed.

Note. boundary (singular) fit: see ?isSingular

Model Results

Fixed Effect Omnibus tests

	X^2	df	p
More readable is human	89.7178	1.00	< .001
Confidence	8.0146	1.00	0.005
Demographics: age	1.8030	1.00	0.179
Demographics: education_ordinal	0.4735	1.00	0.491
Demographics: ai_usage_ordinal	0.0771	1.00	0.781
Demographics: gender merged	0.7445	2.00	0.689
Demographics: field_merged	1.7027	4.00	0.790
Reaction time log	1.1086	1.00	0.292
GLS Euclidean distance	3.7821	1.00	0.052
GLS1 difference	12.4916	1.00	< .001
GLS2 difference	6.2612	1.00	0.012
GLS3 difference	5.0625	1.00	0.024
GLS4 difference	5.7322	1.00	0.017
id	1.1919	1.00	0.275
Demographics: reading_disorder_merged	1.0745	1.00	0.300
GLS8 difference	1.4838	1.00	0.223
GLS7 difference	0.6111	1.00	0.434
GLS6 difference	0.0648	1.00	0.799
GLS5 difference	2.5921	1.00	0.107
Demographics: ai_improve	1.2544	1.00	0.263
Demographics: ai_risk	0.1927	1.00	0.661
Demographics: ai_quality	0.0296	1.00	0.863
Demographics: ai_lacks_creativity	0.0253	1.00	0.874
Demographics: ai_lacks_understanding	1.2504	1.00	0.263
Demographics: ai_grammar	12.8382	1.00	< .001
Demographics: ai_avoidance	1.7876	1.00	0.181
Demographics: ai_dumbing	1.0169	1.00	0.313
Demographics: ai_fascination	0.0266	1.00	0.871

Fixed Effects Parameter Estimates

Names	Effect	Estimate	SE	95% Confidence Interval		exp(B)	95% Exp(B) Confidence Interval		z	p
				Lower	Upper		Lower	Upper		
(Intercept)	(Intercept)	1.07430	0.3503	0.38767	1.76092	2.928	1.474	5.818	3.0666	0.002
More readable is human1	1 - 0	2.06307	0.2178	1.63617	2.48996	7.870	5.135	12.061	9.4719	< .001
Confidence	Confidence	-0.13657	0.0482	-0.23113	-0.04202	0.872	0.794	0.959	-2.8310	0.005
Demographics: age	Demographics: age	0.01868	0.0139	-0.00859	0.04594	1.019	0.991	1.047	1.3428	0.179
Demographics: education_ordinal	Demographics: education_ordinal	0.08890	0.1292	-0.16431	0.34210	1.093	0.848	1.408	0.6881	0.491
Demographics: ai_usage_ordinal	Demographics: ai_usage_ordinal	0.01728	0.0623	-0.10473	0.13929	1.017	0.901	1.149	0.2777	0.781
Demographics: gender merged1	male - female	-0.01249	0.1846	-0.37436	0.34938	0.988	0.688	1.418	-0.0676	0.946
Demographics: gender merged2	other - female	-0.44541	0.5175	-1.45971	0.56890	0.641	0.232	1.766	-0.8607	0.389
Demographics: field_merged1	human - cs	-0.38585	0.3722	-1.11542	0.34372	0.680	0.328	1.410	-1.0366	0.300
Demographics: field_merged2	nature - cs	-0.29247	0.3946	-1.06583	0.48089	0.746	0.344	1.618	-0.7412	0.459
Demographics: field_merged3	other - cs	-0.36696	0.4746	-1.29717	0.56326	0.693	0.273	1.756	-0.7732	0.439
Demographics: field_merged4	philology - cs	-0.51098	0.4184	-1.33099	0.30904	0.600	0.264	1.362	-1.2213	0.222
Reaction time log	Reaction time log	-0.08757	0.0832	-0.25057	0.07544	0.916	0.778	1.078	-1.0529	0.292
GLS Euclidean distance	GLS Euclidean distance	0.09811	0.0504	-7.66e−4	0.19699	1.103	0.999	1.218	1.9448	0.052
GLS1 difference	GLS1 difference	-0.30027	0.0850	-0.46679	-0.13376	0.741	0.627	0.875	-3.5344	< .001
GLS2 difference	GLS2 difference	-0.33332	0.1332	-0.59441	-0.07224	0.717	0.552	0.930	-2.5022	0.012
GLS3 difference	GLS3 difference	0.09330	0.0415	0.01203	0.17458	1.098	1.012	1.191	2.2500	0.024
GLS4 difference	GLS4 difference	0.11974	0.0500	0.02172	0.21776	1.127	1.022	1.243	2.3942	0.017
id	id	-0.01233	0.0113	-0.03447	0.00981	0.988	0.966	1.010	-1.0917	0.275
Demographics: reading_disorder_merged1	true - false	0.71990	0.6945	-0.64126	2.08105	2.054	0.527	8.013	1.0366	0.300
GLS8 difference	GLS8 difference	-0.04195	0.0344	-0.10945	0.02555	0.959	0.896	1.026	-1.2181	0.223
GLS7 difference	GLS7 difference	0.03550	0.0454	-0.05350	0.12450	1.036	0.948	1.133	0.7817	0.434
GLS6 difference	GLS6 difference	0.00881	0.0346	-0.05902	0.07664	1.009	0.943	1.080	0.2545	0.799
GLS5 difference	GLS5 difference	-0.08746	0.0543	-0.19392	0.01901	0.916	0.824	1.019	-1.6100	0.107
Demographics: ai_improve	Demographics: ai_improve	0.08242	0.0736	-0.06181	0.22666	1.086	0.940	1.254	1.1200	0.263
Demographics: ai_risk	Demographics: ai_risk	-0.02545	0.0580	-0.13910	0.08820	0.975	0.870	1.092	-0.4389	0.661
Demographics: ai_quality	Demographics: ai_quality	-0.01152	0.0670	-0.14286	0.11981	0.989	0.867	1.127	-0.1720	0.863
Demographics: ai_lacks_creativity	Demographics: ai_lacks_creativity	-0.01039	0.0654	-0.13853	0.11774	0.990	0.871	1.125	-0.1590	0.874
Demographics: ai_lacks_understanding	Demographics: ai_lacks_understanding	0.07057	0.0631	-0.05313	0.19427	1.073	0.948	1.214	1.1182	0.263
Demographics: ai_grammar	Demographics: ai_grammar	0.23112	0.0645	0.10469	0.35754	1.260	1.110	1.430	3.5830	< .001
Demographics: ai_avoidance	Demographics: ai_avoidance	-0.07768	0.0581	-0.19155	0.03619	0.925	0.826	1.037	-1.3370	0.181
Demographics: ai_dumbing	Demographics: ai_dumbing	0.05286	0.0524	-0.04988	0.15559	1.054	0.951	1.168	1.0084	0.313
Demographics: ai_fascination	Demographics: ai_fascination	-0.01330	0.0816	-0.17320	0.14661	0.987	0.841	1.158	-0.1630	0.871

Random Components

Groups	Name	SD	Variance	ICC
Participant ID	More readable is human0	0.9265	0.85845	
	More readable is human1	1.0687	1.14205	
	Confidence	0.3027	0.09162	
	Reaction time log	0.1454	0.02113	
	GLS Euclidean distance	0.1909	0.03645	
	GLS1 difference	0.4111	0.16902	
	GLS2 difference	0.6810	0.46382	
	GLS3 difference	0.1320	0.01743	
	GLS4 difference	0.1857	0.03448	
	id	0.0494	0.00244	
	GLS8 difference	0.0338	0.00114	
	GLS7 difference	0.1193	0.01423	
	GLS6 difference	0.1179	0.01389	
	GLS5 difference	0.1486	0.02208	
Residuals		1.0000	1.00000	.

Note. Number of Obs: 2244 , groups: Participant ID 132

Random Parameters correlations

Groups	Param.1	Param.2	Corr.
Participant ID	More readable is human0	More readable is human1	-0.1357
	More readable is human0	Confidence	0.7366
	More readable is human0	Reaction time log	-0.5014
	More readable is human0	GLS Euclidean distance	-0.3042
	More readable is human0	GLS1 difference	0.4502
	More readable is human0	GLS2 difference	-0.7730
	More readable is human0	GLS3 difference	-0.1628
	More readable is human0	GLS4 difference	0.1273
	More readable is human0	id	0.8072
	More readable is human0	GLS8 difference	0.6546
	More readable is human0	GLS7 difference	0.5294
	More readable is human0	GLS6 difference	0.0181
	More readable is human0	GLS5 difference	0.1757
	More readable is human1	Confidence	-0.6185
	More readable is human1	Reaction time log	-0.1658
	More readable is human1	GLS Euclidean distance	-0.2715
	More readable is human1	GLS1 difference	-0.3666
	More readable is human1	GLS2 difference	0.1657
	More readable is human1	GLS3 difference	0.1708
	More readable is human1	GLS4 difference	-0.2312
	More readable is human1	id	-0.0489
	More readable is human1	GLS8 difference	0.0591
	More readable is human1	GLS7 difference	-0.2807
	More readable is human1	GLS6 difference	-0.0932
	More readable is human1	GLS5 difference	-0.8651
	Confidence	Reaction time log	-0.1095
	Confidence	GLS Euclidean distance	-0.0626
	Confidence	GLS1 difference	0.6796
	Confidence	GLS2 difference	-0.8108
	Confidence	GLS3 difference	-0.2743
	Confidence	GLS4 difference	0.1229
	Confidence	id	0.5153
	Confidence	GLS8 difference	0.4863
	Confidence	GLS7 difference	0.2745
	Confidence	GLS6 difference	-0.1273
	Confidence	GLS5 difference	0.6892
	Reaction time log	GLS Euclidean distance	0.7467
	Reaction time log	GLS1 difference	0.0868
	Reaction time log	GLS2 difference	-0.0835
	Reaction time log	GLS3 difference	0.4857
	Reaction time log	GLS4 difference	0.5059
	Reaction time log	id	-0.0521
	Reaction time log	GLS8 difference	-0.4404
	Reaction time log	GLS7 difference	-0.6837
	Reaction time log	GLS6 difference	-0.7138
	Reaction time log	GLS5 difference	0.1175
	GLS Euclidean distance	GLS1 difference	-0.0488
	GLS Euclidean distance	GLS2 difference	-0.0866
	GLS Euclidean distance	GLS3 difference	0.6903
	GLS Euclidean distance	GLS4 difference	0.7521
	GLS Euclidean distance	id	0.1383
	GLS Euclidean distance	GLS8 difference	-0.1221
	GLS Euclidean distance	GLS7 difference	-0.4406
	GLS Euclidean distance	GLS6 difference	-0.6644
	GLS Euclidean distance	GLS5 difference	-0.0733
	GLS1 difference	GLS2 difference	-0.6408
	GLS1 difference	GLS3 difference	-0.5700
	GLS1 difference	GLS4 difference	0.3898

Random Parameters correlations

Groups	Param.1	Param.2	Corr.
	GLS1 difference	id	0.1957
	GLS1 difference	GLS8 difference	0.6171
	GLS1 difference	GLS7 difference	-0.2782
	GLS1 difference	GLS6 difference	0.0360
	GLS1 difference	GLS5 difference	0.3820
	GLS2 difference	GLS3 difference	-0.0440
	GLS2 difference	GLS4 difference	-0.3499
	GLS2 difference	id	-0.7701
	GLS2 difference	GLS8 difference	-0.5650
	GLS2 difference	GLS7 difference	0.0128
	GLS2 difference	GLS6 difference	0.5049
	GLS2 difference	GLS5 difference	-0.2486
	GLS3 difference	GLS4 difference	0.3614
	GLS3 difference	id	0.3990
	GLS3 difference	GLS8 difference	-0.3698
	GLS3 difference	GLS7 difference	-0.0685
	GLS3 difference	GLS6 difference	-0.7594
	GLS3 difference	GLS5 difference	-0.3239
	GLS4 difference	id	0.4316
	GLS4 difference	GLS8 difference	0.2738
	GLS4 difference	GLS7 difference	-0.3431
	GLS4 difference	GLS6 difference	-0.4114
	GLS4 difference	GLS5 difference	-0.1346
	id	GLS8 difference	0.3300
	id	GLS7 difference	0.3650
	id	GLS6 difference	-0.4468
	id	GLS5 difference	0.0170
	GLS8 difference	GLS7 difference	-0.0369
	GLS8 difference	GLS6 difference	0.1233
	GLS8 difference	GLS5 difference	-0.1925
	GLS7 difference	GLS6 difference	0.4333
	GLS7 difference	GLS5 difference	0.3075
	GLS6 difference	GLS5 difference	0.0860
